# Toward whole tissue imaging of axolotl regeneration

**DOI:** 10.1002/dvdy.282

**Published:** 2020-12-31

**Authors:** Wouter Masselink, Elly M. Tanaka

**Affiliations:** ^1^ Research Institute of Molecular Pathology (IMP), Vienna BiocCenter (VBC) Vienna Austria

**Keywords:** axolotl, clearing, imaging, regeneration, whole mount

## Abstract

The axolotl is a highly regenerative organism and has been studied in laboratories for over 150 years. Despite a long‐standing fascination with regeneration in general and axolotl specifically, we are still scratching the surface trying to visualize and understand the complex cellular behavior that underlies axolotl regeneration. In this review, we will discuss the progress that has been made in visualizing these processes focusing on four major aspects: cell labeling approaches, the removal of pigmentation, reductionist approaches to perform live cell imaging, and finally recent developments applying tissue clearing strategies to visualize the processes that underly regeneration. We also provide several suggestions that the community could consider exploring, notably the generation of novel alleles that further reduce pigmentation as well as improvements in tissue clearing strategies.

## Key Findings

- Historical perspective on axolotl imaging and lineage tracing
- Description of tissue clearing approaches
- Refractive index matching strategies
- Strategies to further reduce pigmentation in axolotl

## 1 INTRODUCTION

The Axolotl (*Ambystoma mexicanum*) was initially introduced in captivity as a biological curiosity due to its neotenic features. Over the last 150 years however, axolotl has become appreciated as a highly regenerative vertebrate species. Adult axolotls reach over 30 cm in length, are heavily pigmented, and are capable of life‐long regeneration of a wide range of complex tissues and structures including its limbs, brain, spinal cord, heart, and tail. This vertebrate species with such extensive and large‐scale regenerative abilities has inspired generations of researchers to probe the basis of this unique ability.

While at first glance it might seem impossible to perform whole mount imaging on a large and heavily pigmented animal like the axolotl, researchers have successfully tracked cellular fates and behavior. This is possible by taking advantage of various biological aspects, as well as technical innovations including their size invariant regenerative ability, the capacity to generate transgenic animals as well as the existence of mutant animals that are not pigmented. In this review, we will provide an overview of the whole mount imaging that has been done in the axolotl as well as what might still be improved. The recent adaption of tissue clearing and whole mount imaging of fixed tissues provide tissue level views of complex cellular arrangements.

## 2 VISUALIZING CELL MOVEMENT

There has been a long‐standing interest studying the cellular contribution to regenerating appendages. Prior to the development of transgenic animals this was a laborious and challenging approach dependent on the grafting of abnormally pigmented or karyotyped tissue and even using xenografts.^1^, ^2^, ^3^, ^4^ By grafting tissue from wild‐type pigmented axolotl to non‐pigmented mutant axolotls, the contribution of the pigmented epidermis can efficiently be traced.^1^, ^2^, ^4^, ^5^ In these approaches the pigmentation of the epidermis is used as a proxy for the underlying tissue. Xenografts are yet another method of performing lineage tracing through grafting. Tissue grafting between the spotted newt and axolotl is possible, and due to the size difference of these animals, host and donor tissue can be distinguished on a macroscopic level. Newts were also used to perform lineage tracing taking advantage of the size difference of karyotypically abnormal cells.^3^ Haploid cells have a smaller nucleus and can thus be distinguished from diploid host tissue. While these approaches require access to karyotypically abnormal donors the analysis is not restricted to specific pigmented tissues. Karyotype based analysis have also been performed in axolotl. Here triploid axolotl are generated either through heat shock or hydrostatic pressure treatment shortly after fertilization, effectively inhibiting second polar body formation.^6^, ^7^ Using these approaches 30% to 76% of all nuclei can be identified as triploid based on their number of nucleoli. Axolotl are readily amendable to tissue grafting strategies, and as such triploid donor tissue can be grafted in diploid host animals to perform fate mapping and lineage tracing studies.^8^, ^9^, ^10^ When triploid donor tissue is grafted into regenerating diploid host tissue, their contribution to the regenerate can then be determined through serial sectioning, imaging and 3D reconstruction. However by and large, wholemount imaging in the absence of fluorescent markers was limited to large anatomical structures such as the skeleton and superficial structures such as the epidermis.^11^, ^12^, ^13^

Other early attempts at lineage tracing used various labeling strategies ranging from dye labeling approaches to radioactivity and fluorescent plasmid electroporation. Any approach that can label a cell for several divisions could be used to perform at least short‐term lineage tracing experiments. Such transient labeling strategies are intrinsically limited but have been used successfully in various approaches. Of note are [^3^H] thymidine incorporations as well as fluorescent dye injections.^14^, ^15^ [^3^H] thymidine is readily incorporated into the DNA of a donor during cell division. [^3^H] thymidine labeled donor cells are distinguished from host cells after sectioning and autoradiography. Such lineage tracing approaches were first performed in newts but were quickly after that also used to study axolotl regeneration.^16^ These methods, while allowing for more flexibility still are not amendable to whole mount imaging. The first attempts at true whole mount imaging used larvae and took advantage of the size invariant regenerative nature of axolotl. Using the larvae of a non‐pigmented mutant axolotl and either fluorescent dyes injections or electroporation of plasmids encoding fluorescent proteins. Dye labeling approaches were used in the larvae of axolotl tails to image the contribution of muscle fibers to tail regeneration.^15^ In addition dye labeling using calcein effectively allows for whole mount imaging and 3D reconstruction of skeletal structures in vivo.^17^, ^18^ Other work used the electroporation of plasmids to introduce fluorescent labels in small groups of cells. A significant advantage of this approach is that it allows for the co‐electroporation of other biologically active factors, and thus enables in vivo gene function analysis during regeneration. Most notably this approach has been used to study proximo distal patterning in regenerating limbs of axolotl larvae.^19^, ^20^

Grafting and electroporation paradigms allow researchers to perform lineage analysis, fate mapping studies, and study the molecular interactions that control limb regeneration. Imaging of such large tissues is still challenging, limited to either a course analysis of whole mount limbs or tail, or serial sectioning and 3D reconstruction to achieve a high‐resolution analysis. To further improve whole mount imaging quality several aspects including pigmentation, size limitation of the axolotl, and tissue clearing approaches require optimization. In the remainder of this review we will discuss the current state and future directions that have to be explored to further improve these aspects.

## 3 AXOLOTL PIGMENTATION

Wild‐type Axolotls contain a combination of three different pigments in the epidermis Melanophores (black pigment cells), Xanthophores (yellow pigment cells), and Iridophores (silvery pigment cells).^21^ The combination of these three different pigments would prevent most whole mount microscopy analysis. Historically several axolotl pigment mutants have been identified including Melanoid (m) White (d), Albino (a), and Axanthic (Ax).^22^ However by themselves none of these mutants provide robust axolotls devoid of pigmentation. The generation of axolotls devoid of pigmentation would significantly improve the amenability of axolotl to whole‐mount imaging. Melanoid (m) axolotl have an overabundance of melanin, and as the animal matures shows a reduction in xanthophores, while iridophores fail to differentiate completely.^23^ All other mutants show a loss of some pigment cells or pigmentation without increasing overall melanin production making animals more amendable to whole mount imaging.^24^, ^25^ White (d) mutant animals were part of the founding lab strain of axolotls and were found to carry a recessive mutation in endothelin 3 (edn3), disrupting pigment cell migration.^26^ Albino (a) mutant animals were generated through introgression with tiger salamanders which carry a mutation for tyrosinase (tyr^a^). While these animals lack melanin production, this introgression results in a distinct strain of axolotl/tiger salamander hybrids with significant genomic alterations.^26^ While not all axolotl carry tyr^a^, the genomic introgression is endemic to all major laboratory colonies. Other closely related species such as *A*. *velasci* most likely also introgressed with the laboratory axolotl strains. Axanthic (Ax) animals exhibit normal melanophore production, have unpigmented xanthophores, and exhibit a complete failure of iridophore production. While valuable as a resource, axanthic axolotl have poor survivability, likely due to a viral infection primarily in macrophages and pigment cells.^25^

Both albino and white mutants axolotl provide significant improvements for whole mount imaging over existing wild‐type stains and show good survivability in laboratory conditions. However further improvements are likely possible through the careful selection of combinatorial mutants. Similar to zebrafish, axolotls contain melanophores, xanthophores, and iridophores. The development of combinatorial mutants in zebrafish has changed their adult appearance from an animal heavily pigmented, hallmarked by its horizontal stripes to one that is almost transparent. Pigment mutations in zebrafish were identified through forward mutagenesis screens and have resulted in several combinatorial mutants including Casper (Mitfa−/−; mpv17−/−) and Crystal (Mitfa−/−; mpv16−/−; slc45a2−/−).^27^, ^28^, ^29^ While forward mutagenesis screens are impractical in the axolotl, the establishment of CRISPR/Cas9 targeted mutagenesis has opened the possibility of creating mutant axolotl of a similar nature. The effectiveness of such an approach has already been shown in the establishment of the first CRISPR/Cas9 mutant axolotls. In these animals Tyrosinase was mutated, resulting in animals devoid of melanin, effectively phenocopying the existing axolotl albino mutant.^30^ Similar strategies could be used to systematically remove xanthophores and iridophores. The axolotl axanthic mutant lacks xanthophores and is a mono‐allelic recessive trait, however they have poor survivability possibly due to an endemic viral infection. Through a combination of QTL mapping, and genome sequencing it should be possible to identify the allele responsible for the axanthic phenotype. Subsequently phenocopying this through CRISPR/Cas9 mutagenesis is likely to phenocopy the axanthic mutant phenotype without negatively affecting survival. White;Tyr^−/−^ combinatorial mutant axolotls are our current preferred choice to perform live cell imaging in both embryonic and adult stages. Alternative strategies could be based on the targeted mutagenesis of known pigmentation‐associated genes, in particular the previously mentioned zebrafish pigmentation mutants, slc45a2, mitfa, and mpv17. These genes all have homologues in axolotl and provide a valuable list of additional targets. The systemic disruption of axolotl pigmentation through CRISPR/Cas9 targeted mutagenesis has the potential to result in combinatorial mutant axolotl devoid of pigmentation. This would further improve upon the existing white and Tyr^−/−^ mutants and significantly improve whole mount imaging during limb and tail regeneration. Additionally, it would open up access to organs that due to pigmentation are otherwise inaccessible such as the eye and brain, but also significantly improve imaging for embryonic studies. Future work through combinatorial approaches as well as the generation of novel mutant alleles should allow us to generate axolotls completely devoid of pigmentation.

## 4 LIVE CELL IMAGING IN AXOLOTL

Until the establishment of germline modification and transgenesis, lineage tracing and imaging in axolotls was limited to triploid cell grafting, dye labeling, or electroporation‐based approaches. The development of germline modification and transgenesis in the axolotl have made it possible to study gene activity, perform lineage analysis, and study positional identity during regeneration in unprecedented detail.^31^, ^32^, ^33^, ^34^ Grafting experiments using ubiquitous GFP expressing axolotl provide several unique advantages over previous grafting strategies. Most notably the ease of visualizing grafted tissue on both macro and microscopic levels. Grafting studies using ubiquitous GFP expressing donor tissue have provided further insight into the precise origin of cells that contribute to the blastema as well as the lineage potential of specific tissues.^31^, ^34^, ^35^ Other more complex strategies have used a transgenic cassette that encodes for several different fluorophores, random recombination results in a range of unique colors to be present in individual cells. This can then be used to perform clonal analysis, both in the context of time‐lapse microscopy as well as retrospectively.^18^, ^36^ These developments have radically improved the ability to perform live cell imaging in regenerating axolotl and can be used to uncover important biological insights.

Despite the development of transgenic axolotls live cell imaging remains a challenge due to the size of adult axolotls. Due to the neotenic nature of the axolotl, the overall body plan is laid out at a relative early stage when axolotls are under 2 cm in length. At this point all major features of the adult body plan such as its tail, limbs, gills, and cranio‐facial features are already specified. This is followed by a relatively long period of continued growth, reaching a maximum of 30 cm in length for adult axolotl. In order to capture regenerative events in such a large adult animal, large volumes would have to be imaged for a long duration, which presents serious technical difficulties. However, we would like to argue that by taking a reductionist approach it is possible to capture fundamental cell dynamic processes that underlie regeneration regardless the amputation plane or size of the animal. This argument is based on two premises. First, size invariant regeneration employs similar mechanisms regardless of the size or age of the animal. Second, the cell dynamic processes that control regeneration are conserved regardless of the amputation plane.

Size invariant regeneration is a well‐established phenomenon in axolotl. Axolotl have lifelong regenerative capacity and manage to regenerate limbs that were artificially reduced in size.^37^ In this study Bryant and colleagues performed repeated removal of axolotl limb buds, resulting in artificially size reduced limbs. Upon amputation these limbs were found to regenerate the correct size. While the mechanisms which underlies this phenomenon have not been directly addressed, size invariant patterning during regeneration and development has been studied on a molecular level in a variety of organisms including hydra, zebrafish, and planaria. In these studies authors consistently find that the scaling of patterning follows a concomitant scaling of cellular and molecular mechanism. As such we can infer that as long as patterning occurs correctly, the mechanisms of regeneration are likely comparable regardless the size of the animal. Using small juvenile axolotls is therefore a viable strategy to perform live cell imaging studies during axolotl regeneration.

Size invariant regeneration seems to be conserved not just in axolotl but across salamanders. In newt both tail and limbs go through distinct morphological steps during regeneration, regardless their amputation plane.^38^, ^39^, ^40^ It should be noted though that proximal and distal amputations take a comparable amount of time to regenerate, so obvious differences in proliferation, specifically the rate at which cells withdraw from a proliferative phase, can differ significantly between amputation planes.^40^ Even when comparing two extreme examples, digit and limb regeneration, the same cellular processes were found to be present during regeneration.^18^, ^36^ Currie et al used live cell imaging of the regenerating digit to reveal that fibroblasts contribute to all connective tissue lineages. On the other hand Gerber et al, used single cell RNA sequencing of limb regeneration to come to a similar conclusion. Taken together this would suggest that regeneration occurs through similar processes regardless of the amputation plane. Care should be taken interpreting results from extremely distal amputations as the relative proportion of tissues at those levels can differ significantly from more proximal amputation planes. For example muscle tissue is virtually absent in digits. Live cell imaging in a radically reductionist approach such as digit regeneration, can be informative for the processes that control the regeneration of larger structures such as limbs.

The refractive index mismatch between the immersion medium (often water) and the biological specimen leads to a reduction in image quality. By matching the refractive index of the immersion medium closely with the refractive index of the specimen, better resolution and signal‐to‐noise ratios at greater tissue depths can be achieved. Boothe et al identified Iodixanol as a supplement to tune the refractive index of water‐based media for a wide range of organisms.^41^ Commercially available under the brand name Optiprep, Iodixanol is compatible with living cells and tissues. It provides a cheap and simple strategy to improve the effective imaging depth in many aquatic organisms including axolotl. Novel developments in the understanding of cephalopod skin have opened up the exciting opportunity to begin to tune the refractive index of tissue in vivo.^42^

While mature axolotls are large animals not well suited to live cell imaging, several strategies can be used to image cellular dynamics of regeneration. By employing a combination of small, juvenile axolotls, distal amputations, and refractive index matching through approaches such as iodixanol, live cell imaging during axolotl regeneration is feasible.

## 5 TISSUE CLEARING

Tissue clearing is the process of creating homogeneous refractive indexes within a tissue, in effect turning a previously turbid and opaque tissue transparent. In contrast to refractive index tuning of the immersion medium with Iodixanol, Tissue clearing is performed on fixed tissue and changes the refractive index of the tissue. Turbidity and opaqueness are inherent to biological tissues due to the refractive index mismatch between a tissues three constituent components, namely lipids (RI 1.4), water (RI 1.3), and protein (RI 1.5). By homogenizing the refractive index within a tissue and matching this to the refractive index of the immersion liquid, tissues can be made transparent. A wide range of tissue clearing methods have been developed, predominantly focusing on the clearing of brain tissue in order to collect information on long axonal projections which would be lost in sectioned tissue. However these principles can be applied broadly and should provide a powerful approach imaging any large and complex tissues. Dehydration based clearing strategies were first developed by Spalteholz and adapted to axolotl using oil of wintergreen as a clearing agent.^43^, ^44^, ^45^ More recently we have seen the development and implementation of several clearing strategies on axolotl. Methods such as 2Eci and Salamander‐Eci were found to efficiently clear axolotl tissues, preserve endogenous fluorescence and are compatible with dye labeling strategies such as Sytox Green.^46^, ^47^ We have already taken advantage of this approach and used it to study the complex cellular processes and clonal dynamics during axolotl limb regeneration.^36^ This approach is particularly powerful when dealing with either fluorescent protein expression or antibody labeled tissues. 2Eci has since become a standard in the daily operation of our lab and is used to study the regeneration of brain, spinal cord, limb, and tail.

While 2Eci is particularly suited to the clearing of tissues labeled either with endogenous fluorescent proteins or antibodies, other methods might prove better suited to the clearing of RNA Fluorescent In‐Situ Hybridization (FISH) labeled tissues. Labeled samples are stored in ethanol and are dehydrated. As such any RI matching media that preserves the fluorescent signal should prove effective. Several mounting media such a glycerol as well as TDE have proven to be an effective clearing agent of FISH labeled samples. Indeed TDE based strategies have been successfully used by Duerr and colleagues to visualize nascent synthesis of various biomolecules including DNA, RNA, and protein.^48^ Dehydration based tissue clearing approaches generally suffer from a mild increase in auto‐fluorescence, as such labeling approaches such as single molecule HCR‐FISH are best done using hydration‐based approaches. More recently a novel hydration clearing approach by Pende and colleagues was shown to be effective in depigmenting and clearing tissues while also allowing for the imaging of FISH and antibody labeled tissues.^49^ The continued development and optimization of clearing and imaging strategies suited to axolotl will provide an unprecedented ability to image the complex process of regeneration.

## 6 FUTURE DIRECTIONS

From the early days of axolotl as a laboratory animal over a 150 years ago to the present day, axolotl research has radically transformed. Starting as a weird and curious animal, studied for its neoteny, more recently axolotl have seen use as a model to study stem cells and regeneration. Over this time technological advances in genetic manipulation, microscopic imaging, and tissue clearing have transformed the axolotl from a curious heavily pigmented mystery to a system in which individual cells can now be traced and complex in vivo interactions recorded.

We expect that the continued development of mutant and transgenic lines will eventually result in the complete removal of all skin pigmentation in the axolotl, further improving the ability to probe deep inside the complex process of regeneration in vivo. Furthermore, the further development of clearing approaches with reduced autofluorescence background signal should allow even weak fluorescent signals to be imaged. Finally, the continued advancement of light sheet microscopes and their adaption to the peculiarities of axolotl biology, will provide continued improvements to the field.

## AUTHOR CONTRIBUTIONS

**Wouter Masselink:** Conceptualization; investigation; project administration; writing‐original draft; writing‐review and editing. **Elly Tanaka:** Conceptualization; project administration; supervision; writing‐original draft.
